# Research status and prospects of molecular pathological mechanisms and novel therapeutic targets of osteosarcoma: a systematic review

**DOI:** 10.3389/fonc.2025.1665299

**Published:** 2026-01-20

**Authors:** Huan Jin, Cai Huang, Ying Dong, Qi Xiong, Di Wang, Ziyi He, Yu Shang, Lin Shen, Chen Ma, Zixian Wang, Siwei Shi, LingFeng Zeng, Bo Shuai

**Affiliations:** 1 Department of Integrated Traditional Chinese and Western Medicine, Union Hospital, Tongji Medical College, Huazhong University of Science and Technology, Wuhan, China; 2 College of Sports Medicine, Wuhan Sports University, Wuhan, China; 3 Department of Internal Medicine, Rongjun Hospital of Hubei, Wuhan, China; 4 Guangdong Provincial Academy of Chinese Medical Sciences, Guangzhou University of Chinese Medicine, Guangzhou, China

**Keywords:** cutting-edge technology, multi-omics research, osteosarcoma, pathological mechanisms, targeted therapy

## Abstract

**Background:**

The treatment of osteosarcoma, a highly aggressive primary malignant bone tumour, has long faced limitations due to chemotherapy resistance, tumour het-erogeneity, and an immunosuppressive microenvironment.

**Methods:**

This review synthesizes recent multi-omics and clinical trial data to analyse synergistic oncogenic mechanisms in osteosarcoma—including driver mutations (TP53/RB1), epigenetic re-programming (m^6^A/ncRNA networks), and dysregulated pathways (PI3K/AKT, Wnt/β-catenin)—and evaluates derived therapeutic strategies.

**Results:**

Targeted therapies demonstrate potential to improve prognosis in clinical trials; immunotherapies significantly enhance response rates by remodelling the cold tumour microenvironment; advanced technologies like nanotechnology and 3D-printed scaffolds over-come limitations of conventional treatments and enable integrated diagnosis and therapy. However, tumour evolutionary heterogeneity, off-target toxicity of targeted therapies, and translational gaps between animal models and clinical efficacy remain major challenges.

**Conclusions:**

Future directions require integrating AI-driven imaging omics, spatiotemporal multi-omics, and mechanically adaptive biomaterials to establish a precision management system. This will advance osteosarcoma therapy from survival prolongation toward functional cure—defined as complete tumour eradication with physiological reconstruction of bone structure/function (e.g., restoring load-bearing/joint mobility), while preventing treatment-related disability, ultimately achieving oncologic cure with preserved quality of life.

## 1 Introduction

As the most common primary malignant bone tumour, osteosarcoma (OS) has an annual incidence of 3.4–4.2 cases per million people worldwide, with significant regional differences. The incidence rate in Asia (4.5–5.1 per million) is higher than that in Africa (2.8–3.2 per million) and Europe (3.6–4.0 per million), and the survival rate in developing countries is generally lower than that in developed countries (the five-year survival rate difference is 15–20%) (1). Notably, this tumour tends to occur in the long diaphyseal epiphyses of children and adolescents (peak age 10–25 years, accounting for 75% of all cases); however, recent epidemiological data show that the age distribution of OS in the United States has shifted from classic bimodal (adolescents and older adults) to unimodal (concentrated in adolescents), which may be related to environmental exposure or changes in diagnostic patterns (2). Its aggressive and early metastatic (especially lung metastasis) biological characteristics lead to a five-year survival rate of approximately 60–70% in cases without metastasis, while it plummets to <20% in cases with metastasis or recurrence (3). Although surgery combined with neoadjuvant chemotherapy (such as methotrexate, cisplatin, and doxorubicin) significantly improves the local control rate, chemotherapy resistance, tumour heterogeneity, and microenvironment remodelling remain the main bottlenecks in improving clinical efficacy.

In recent years, breakthroughs in high-throughput sequencing technology and multi-omics analysis have gradually elucidated the molecular pathological mechanisms underlying the abnormal activation of OS driver gene mutations (e.g., inactivation of TP53 [70% of cases] and RB1 [35% of cases]), chromosomal instability, epigenetic disorders (e.g., non-coding RNA network dysregulation), and key signalling pathways (Wnt/β-catenin, PI3K/AKT/mTOR). This stands in sharp contrast to bone malignancies such as Ewing sarcoma, which are primarily driven by chromosomal translocations (e.g., EWS–FLI1 fusion), show a low frequency of TP53/RB1 mutations (<10%), and exhibit significantly greater genomic stability than OS (4, 5). This molecular divergence directly contributes to differing treatment responses; OS shows reduced sensitivity to DNA damage-based chemotherapy (e.g., cisplatin), whereas Ewing sarcoma presents a specific therapeutic window through targeting EWS-FLI1-mediated transcriptional regulatory networks (6). Additionally, regarding metabolic characteristics, OS depends on both glycolysis and oxidative phosphorylation (regulated by RNA acetylation via NAT10), while Ewing sarcoma is more reliant on glutamine metabolism (7), providing a rationale for the development of tailored metabolic targets.

However, the complexity of the molecular typing of OS, the infiltration of immunosuppressive cells (such as M2 tumour-associated macrophages) into the tumour microenvironment, and the lack of targeted drug penetration make it challenging for monotherapy to achieve long-term remission. For instance, bevacizumab targeting VEGF can inhibit angiogenesis; however, its efficacy is limited by compensatory bypass activation. The response rate to PD-1/PD-L1 inhibitors in cold tumour microenvironments is <20% (8). Therefore, the analysis of the dynamic regulatory network governing the interaction between tumour cells and the microenvironment, as well as the design of combined therapeutic strategies based on this (such as target-immune coordination and nanodrug delivery systems), have become key directions to overcome the current therapeutic dilemma.

This study systematically reviews recent advances in the molecular pathological mechanisms of OS, with a focus on the roles of driver gene mutations, epigenetic reprogramming, and immune escape in promoting malignant phenotypes. Moreover, it examines the transformation potential of targeted therapy, cell therapy, and advanced technologies, and proposes multidisciplinary collaborative innovation strategies to overcome the challenges in clinical translation, offering a reference for constructing a precise OS treatment system.

A literature search was conducted in PubMed, Web of Science, and Embase using the terms “osteosarcoma” AND (“molecular mechanisms” OR “signalling pathways” OR “genomic alterations” OR “epigenetic regulation” OR “tumour microenvironment” OR “targeted therapy” OR “immunotherapy”), covering publications from 2014 to 2024. Studies were considered eligible if they focused on osteosarcoma-related molecular mechanisms or therapeutic targets, included original research or review articles, involved human data or relevant preclinical (cell or animal) models, and were published in English. Articles unrelated to OS or lacking mechanistic or biological relevance to OS pathogenesis or treatment were excluded.

## 2 Genomic abnormalities in OS

**Figure 1:**
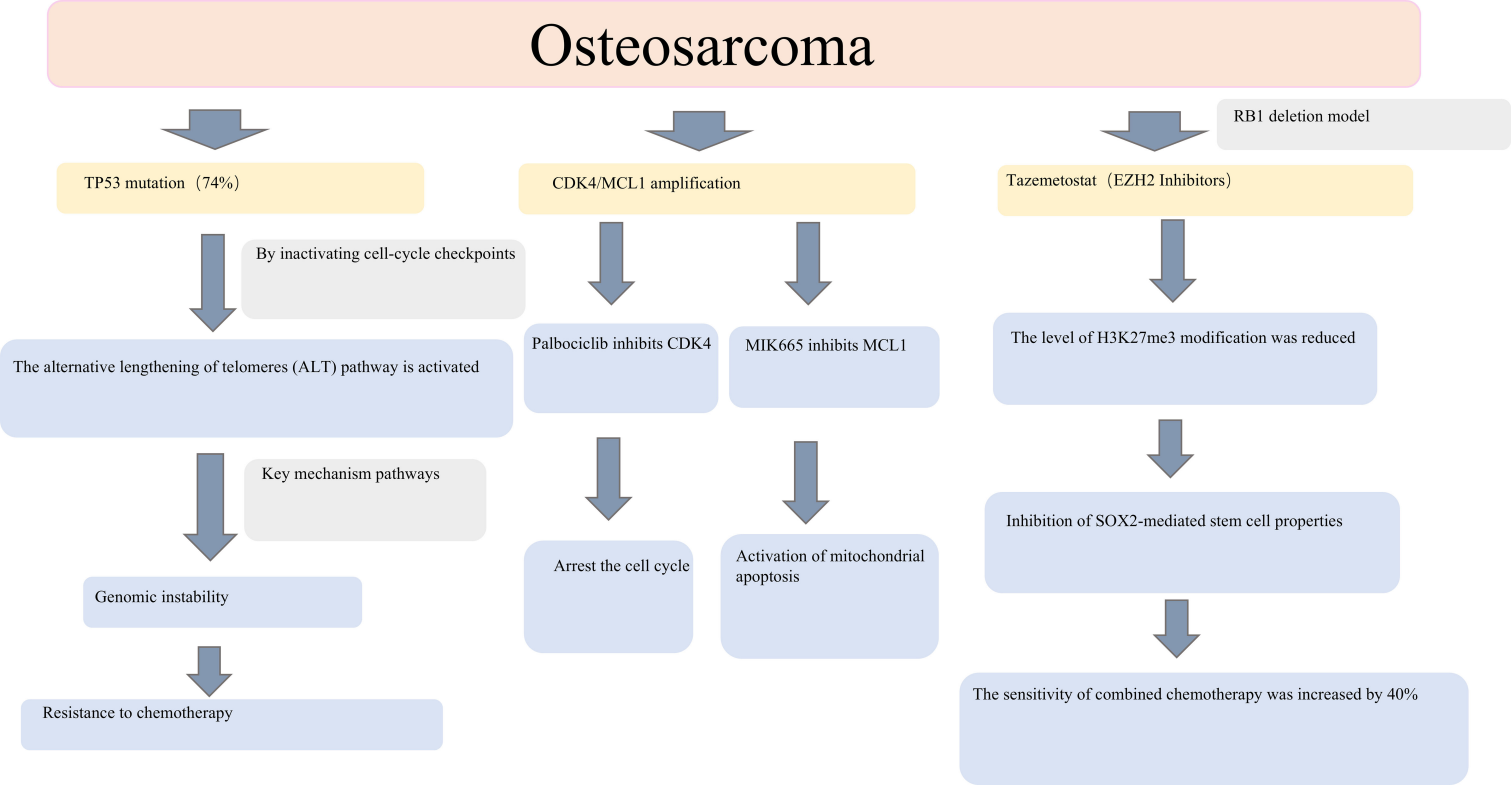
Molecular pathological mechanisms and targeted strategies in osteosarcoma. This schematic illustrates the molecular mechanisms underlying osteosarcoma. Key features include *TP53* mutations driving the activation of the alternative lengthening of telomeres (ALT) pathway and chemotherapy resistance, CDK4/MCL1 amplification, synergistic targeted inhibition strategies, and the mechanism by which the EZH2 inhibitor tazemetostat enhances chemosensitivity in RB1-deficient models. Collectively, these data provide molecular insights into osteosarcoma research and therapeutic development.

OS is a highly malignant tumour characterised by profound genomic instability, which represents a central driving force underlying tumour initiation, progression, therapeutic response, and clinical outcome. Rather than being driven by recurrent single-gene mutations, OS exhibits extensive inter- and intratumoural heterogeneity. Its core oncogenic mechanism is dominated by a synergistic network of structural variations and somatic copy number alterations (SCNAs), which collectively orchestrate clonal evolution, treatment resistance, and disease aggressiveness (**Figure 1**).

Among these alterations, TP53 mutation, present in approximately 74% of OS cases, constitutes a key initiating event that drives genomic instability. Loss of TP53 function promotes activation of the alternative lengthening of telomeres (ALT) pathway, leading to telomere dysfunction, chromosomal fusion, and catastrophic genome rearrangements. This TP53 mutation–ALT axis establishes a permissive genomic landscape for clonal diversification and is closely associated with resistance to DNA-damaging chemotherapeutic agents (TP53 mutation → ALT activation → telomere crisis → chromosomal fusion → chemotherapy resistance) (9). Beyond its canonical tumour-suppressive role, TP53 loss or mutation profoundly reshapes the tumour immune microenvironment. Mutant TP53 impairs antigen processing and presentation by downregulating MHC class I–related machinery and suppressing innate immune sensing pathways such as cGAS–STING, leading to reduced CD8^+^ T-cell and NK-cell infiltration. In parallel, TP53 dysfunction promotes immunosuppressive cytokine signalling and chronic inflammation, thereby facilitating immune escape and resistance to immunotherapy (10).

Concurrently, RB1 deletion (≈16%) cooperates with focal oncogene amplifications to reinforce malignant proliferation and survival. Amplification of CDK4 (≈46%) accelerates G1/S cell-cycle transition, while co-amplification of MYC (≈70%) and the anti-apoptotic factor MCL1 (≈70%) suppresses Bax/Bak-mediated mitochondrial apoptosis. Collectively, these SCNA-driven alterations establish a coordinated proliferation–anti-apoptosis axis that enables sustained tumour growth and survival under therapeutic stress (9).

Importantly, beyond their canonical roles in regulating cell-cycle progression and apoptosis, these key driver genes are increasingly recognized as critical modulators of the tumour microenvironment. RB1 alterations shape the immune landscape by modulating immune cell infiltration, engaging innate immune sensing pathways such as cGAS/STING, and regulating pro-inflammatory chemokines, thereby influencing the magnitude and quality of antitumour immunity (11). Similarly, aberrant activation of the CDK4/6 axis promotes immune evasion by suppressing antigen presentation and interferon responses, whereas pharmacological CDK4/6 inhibition enhances MHC class I expression, promotes cytotoxic T-cell activation, and alleviates regulatory T-cell–mediated immunosuppression, providing a mechanistic basis for synergy with immune checkpoint blockade (12). Beyond its canonical oncogenic functions, MYC has emerged as a central determinant of the tumour immune microenvironment. Aberrant MYC activation suppresses antigen presentation and interferon signalling while upregulating immune checkpoints such as PD-L1 and CD47, thereby promoting immune evasion. In OS, high c-Myc expression is associated with reduced T-cell infiltration and an immune-cold phenotype, contributing to primary resistance to immune checkpoint blockade (13). Accordingly, pharmacological inhibition of MYC can reprogramme the immune microenvironment by enhancing T-cell recruitment and activation, providing a mechanistic rationale for MYC-targeted–immunotherapy combination strategies (14). In parallel, MCL1 amplification or overexpression, classically linked to apoptosis resistance, has emerged as a modulator of the tumour microenvironment. Elevated MCL1 expression correlates with immunosuppressive immune cell infiltration patterns and altered macrophage polarization, collectively favouring an immune-tolerant niche and potentially limiting the efficacy of immune-based therapies (15).

Together, these mechanistic insights provide a compelling rationale for genome-matched therapeutic strategies targeting SCNA-driven oncogenic dependencies. In preclinical models, pharmacological inhibition of CDK4 with palbociclib suppresses more than 60% of tumour growth, while targeting the amplified survival pathway using the MCL1 inhibitor MIK665—particularly in combination with insulin-like growth factor-1 receptor (IGF-1R) blockade—induces marked tumour regression in patient-derived tumour xenograft models (16). These findings underscore the therapeutic vulnerabilities created by SCNA-driven oncogene addiction and highlight opportunities for rational combination strategies.

Importantly, OS exhibits dynamic genomic evolution during disease progression and under treatment pressure. Compared with primary tumours, metastatic lesions demonstrate a 1.68-fold increase in GPC3 mutations, resulting in aberrant activation of the Wnt/β-catenin pathway and enhanced metastatic potential (17). In parallel, enrichment of CDK4-amplified subclones in metastatic sites reflects clonal selection during progression, highlighting the adaptive and evolutionary nature of SCNA-driven tumour heterogeneity (16). Building on these observations, emerging evidence suggests that metastatic subclones driven by CDK4 amplification may remain sensitive to CDK4 inhibition, whereas relapsed or treatment-resistant lesions frequently acquire additional alterations, such as MCL1 copy-number gains, necessitating rational combination strategies incorporating MCL1 inhibitors and IGF-1R blockade. These data further support an adaptive treatment paradigm guided by evolving SCNA profiles (18, 19).

Beyond intrinsic tumour cell survival, specific SCNAs also intersect with therapeutic resistance through modulation of the tumour microenvironment. FGFR1 amplification is significantly associated with chemotherapy resistance, and combined targeting with the FGFR1/VEGFR2 inhibitor sulfatinib and the CSF1R inhibitor pexidartinib has been shown to synergistically suppress immunosuppressive M2-polarised tumour-associated macrophages (TAMs) and myeloid-derived suppressor cells (MDSCs), thereby linking genomic alterations to microenvironmental remodelling and immune suppression (9, 20).

Collectively, these findings indicate that SCNA-driven genomic instability in OS functions not only as an initiating oncogenic force but also as a dynamic regulator of clonal evolution, therapeutic sensitivity, immune contexture, and resistance development, underscoring the necessity of mechanism-guided, genome-informed precision therapy.

## 3 Epigenetic regulatory imbalance in OS

**Figure 2:**
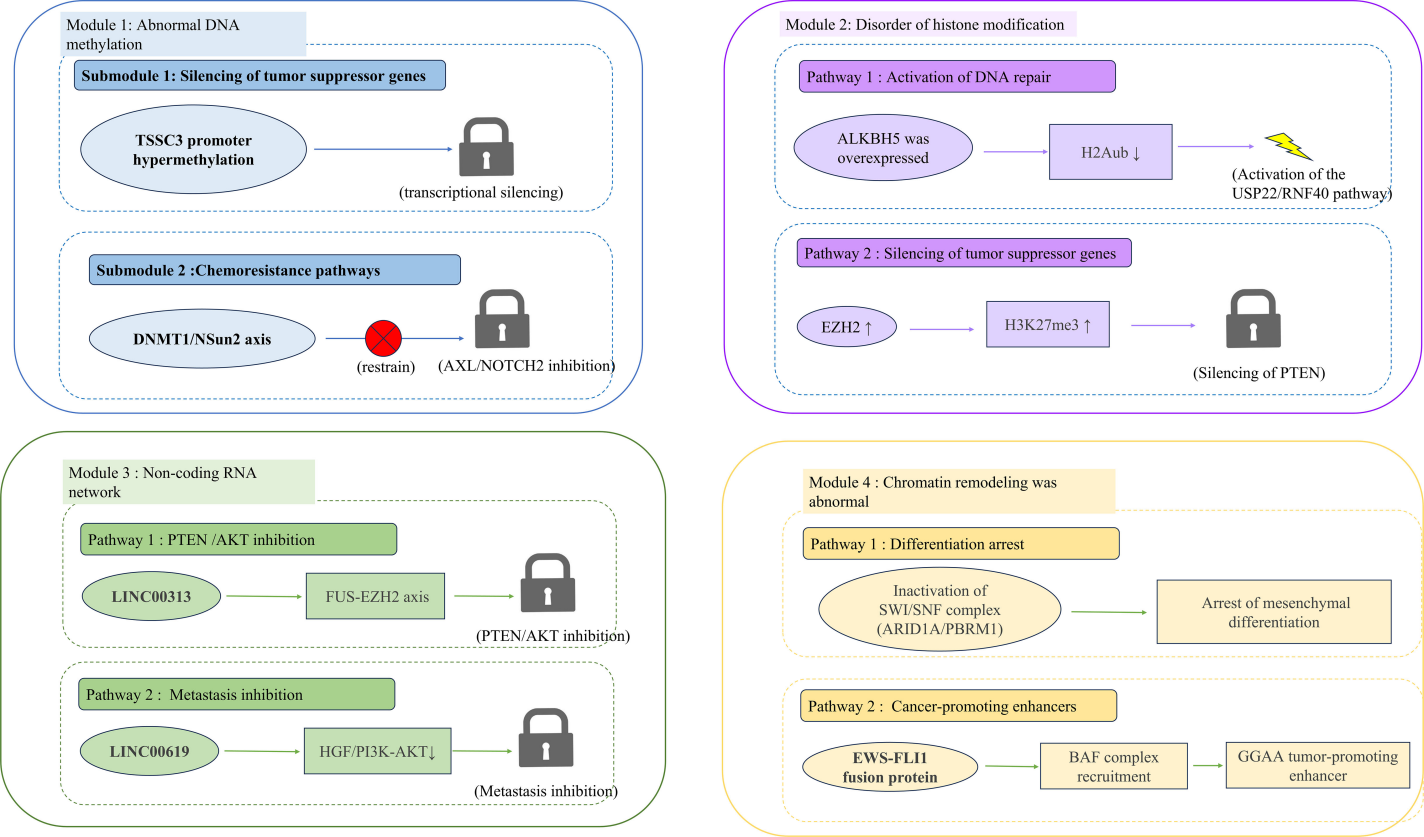
Epigenetic regulatory mechanisms in osteosarcoma. Module 1. DNA Hypermethylation Drives the Silencing of Tumour Suppression and Induces Chemoresistance: TSSC3 promoter hypermethylation (blue hexagon) leads to the transcriptional silencing of this tumour suppressor gene. The DNMT1/NSun2 methylation axis (red inhibitory arrow) suppresses the expression of the anti-apoptotic genes AXL and NOTCH2, thereby contributing to resistance to chemotherapy. Module 2. ALKBH5 Overexpression and EZH2-Mediated Epigenetic Addiction: ALKBH5 overexpression (purple hexagon) reduces H2A ubiquitination (H2Aub↓), activating USP22/RNF40-dependent DNA repair pathways (green arrow). EZH2 upregulation (purple hexagons) increased H3K27me3 levels and silenced PTEN (red inhibitory arrows). The EZH2 inhibitor tazemetostat (pill icon) exhibits synthetic lethality in the SWI/SNF-deficient subtypes. Module 3. LINC00313 and LINC00619 as Key Regulatory RNAs: LINC00313 stabilises EZH2 mRNA via the FUS–EZH2 axis (red dashed arrow), thus inhibiting the PTEN/AKT pathway. LINC00619 suppressed metastasis by downregulating HGF/PI3K–AKT signalling (green dashed arrow). Module 4. WI/SNF Inactivation and EWS-FLI1-Driven Enhancer Activation: SWI/SNF complex inactivation (ARID1A/PBRM1 loss, diamond shape) blocks mesenchymal differentiation. The EWS–FLI1 fusion protein recruits the BAF complex to GGAA microsatellite regions, activating pro-tumorigenic enhancers (dark blue arrow).

The occurrence and progression of OS are driven not only by extensive genomic abnormalities but also by pervasive dysregulation of epigenetic regulatory networks, which act as a critical intermediary linking genetic instability to transcriptional plasticity, immune modulation, and therapeutic resistance (**Figure 2**). These epigenetic alterations involve multiple interconnected mechanisms, including DNA methylation, histone modification, RNA epigenetics, non-coding RNA regulation, and chromatin remodelling.

Aberrant DNA methylation represents a fundamental epigenetic mechanism contributing to OS progression. Hypermethylation of tumour suppressor gene promoters, such as TSSC3, leads to stable transcriptional silencing and promotes tumour survival. Beyond canonical DNA methylation, DNMT1-mediated NSun2 methylation establishes DNA–RNA methylation interaction networks that cooperatively repress apoptosis- and differentiation-related genes, including AXL and NOTCH2, thereby exacerbating chemotherapy resistance and clonal persistence under therapeutic pressure (21, 22). These findings illustrate how DNA methylation abnormalities amplify treatment resistance downstream of genomic instability.

Disruption of histone modification homeostasis further reinforces epigenetic dependency in OS. Overexpression of the m^6^A demethylase ALKBH5 inhibits H2A monoubiquitination (H2Aub), leading to activation of the USP22/RNF40-dependent DNA repair pathway and enhanced tolerance to genotoxic stress. Expansion of ALKBH family demethylases induces an imbalance in histone ubiquitination, resulting in tumour-specific epigenetic addiction and sustained malignant phenotypes (23, 24).

Among histone methylation regulators, EZH2-mediated H3K27me3 modification emerges as a central epigenetic hub integrating oncogenic signalling, lineage plasticity, and immune regulation. Aberrant EZH2 upregulation silences tumour suppressor genes such as PTEN, thereby activating the PI3K/AKT pathway and promoting metastasis. Pharmacological inhibition of EZH2 with tazemetostat not only reduces global H3K27me3 levels but also induces synthetic lethality in SWI/SNF complex-deficient OS subtypes (25, 26). Notably, EZH2 inhibition enhances the therapeutic efficacy of anti-GD2 antibody–drug conjugates, providing a mechanistic rationale for epigenetic–immunotherapeutic combination strategies (27).

Non-coding RNA networks constitute another critical layer of epigenetic regulation in OS. The long non-coding RNA LINC00313 stabilizes EZH2 mRNA via the FUS–EZH2 axis, suppresses PTEN expression, and activates AKT signalling, thereby promoting metastasis and drug resistance. In contrast, LINC00619 suppresses tumour metastasis by regulating HGF/PI3K–AKT signalling, highlighting the context-dependent roles of lncRNAs as either oncogenic drivers or tumour suppressors and underscoring their potential as therapeutic targets (28, 29).

Abnormal chromatin remodelling further contributes to transcriptional reprogramming in OS. Inactivation of the SWI/SNF complex components ARID1A and PBRM1 impairs mesenchymal differentiation and promotes malignant dedifferentiation, while oncogenic fusion proteins recruit the BAF complex to GGAA microsatellite regions via prion-like domains, activating tumour-promoting enhancer landscapes and reinforcing epigenetic addiction (30, 31).

Importantly, epigenetic dysregulation directly shapes the immune microenvironment and therapeutic responsiveness in OS. The DNMT1 inhibitor decitabine enhances CD8^+^ T cell infiltration by reversing CXCL12 promoter methylation and significantly suppresses lung metastasis in OS with low CXCL12 expression, illustrating how epigenetic therapy can reprogram an immunosuppressive tumour microenvironment (32). Consistently, prognostic models based on epigenetic regulators such as SFMBT2 and SMARCA4 effectively predict immune microenvironment characteristics and responses to PD-1 inhibitors, supporting the clinical relevance of epigenetic biomarkers in immunotherapy stratification (33).

These mechanistic insights have accelerated translational advances in epigenetic therapy. Current clinical trials are evaluating combinations of histone deacetylase inhibitors (e.g., vorinostat) with immune checkpoint inhibitors, while preclinical studies are exploring small-molecule drugs targeting m^6^A regulators (METTL3/ALKBH5) and antisense oligonucleotides against oncogenic lncRNAs such as LINC00313 (34–37). This evolution marks a shift in epigenetic therapy from single-target inhibition toward modulation of multidimensional regulatory networks.

Nevertheless, the functional balance of epigenetic regulators remains critical. METTL3-mediated m^6^A modification drives metastasis through activation of YAP signalling, highlighting the therapeutic importance of restoring homeostasis between m^6^A “writer” and “eraser” enzymes rather than indiscriminate inhibition (38). In parallel, a prognostic model based on RNA adenosine modification-related genes (AUC = 0.86) demonstrated significant tumour growth suppression by inhibiting Smad3-dependent cell cycle progression (39).

Additional histone modification pathways further expand the therapeutic landscape. Phosphorylation of CBX4 at T437 promotes lung metastasis by recruiting GCN5 to maintain H3K27 acetylation at the Runx2 promoter, whereas the FDA-approved CK1α activator pirividone induces CBX4 degradation and reduces metastasis by 58% in patient-derived xenograft models (40). In addition, DNA methylation regulators such as 5-azacytidine enhance tumour immunogenicity by activating endogenous retroviruses, offering new opportunities for epigenetic–immune combination therapy (41).

In summary, epigenetic dysregulation in OS functions as a central mediator connecting genomic instability to transcriptional reprogramming, immune evasion, and therapeutic resistance. Precise modulation and restoration of homeostatic control across key epigenetic nodes—including RNA modification, histone modification, non-coding RNAs, and chromatin remodelling—represent promising strategies to advance OS treatment with improved efficacy and reduced toxicity.

## 4 Misalignment of key signal pathways in OS

**Figure 3:**
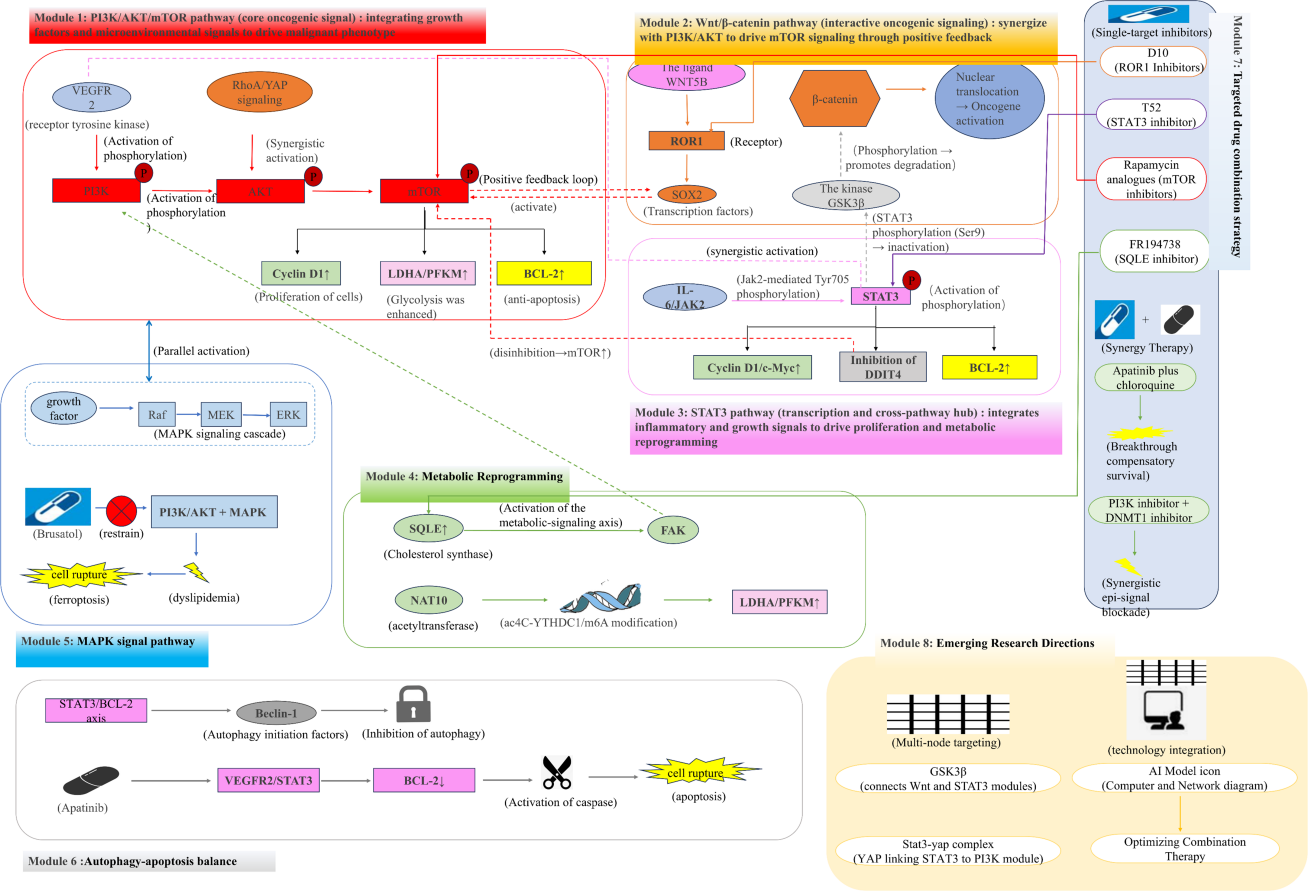
Hierarchical signalling networks and therapeutic translation in osteosarcoma. Part I. Core Signalling Modules 1–3: 1. PI3K/AKT/mTOR pathway (red): Integrates upstream signals (VEGFR2, RhoA/YAP) to drive proliferation (Cyclin D1↑), glycolysis (LDHA/PFKM↑), and anti-apoptosis (BCL-2↑). 2. Wnt/β-catenin pathway (orange): Activated by the WNT5B–ROR1–SOX2 axis, synergising with PI3K/AKT via mTOR and β-catenin stabilisation (GSK3β inhibition by STAT3). 3. STAT3 pathway (purple): The activation of IL-6/JAK2 and VEGFR2 promotes cell cycle progression (Cyclin D1/c-Myc), mTOR activation (via DDIT4 suppression), and BCL-2-mediated survival. Part II. Cross-Regulatory Modules 4–6: 4. Metabolic reprogramming (green): SQLE-driven cholesterol synthesis activates FAK/PI3K/AKT/mTOR axis, while NAT10-mediated ac4C-YTHDC1/m6A epitranscriptomics enhance glycolytic enzymes (LDHA/PFKM↑). 5. MAPK pathway (blue): Similar to the PI3K/AKT pathway, brusatol disrupts lipid metabolism and induces ferroptosis. 6. Autophagy-apoptosis balance (grey): The STAT3/BCL-2 axis suppresses Beclin-1-dependent autophagy, and apatinib inhibits VEGFR2/STAT3 to activate caspase-mediated apoptosis. Part III. Clinical Translation Modules 7–8: 7. Targeted therapies: Single-agent inhibitors (ROR1i-D10, STAT3i-T52, mTORi-rapalogs, and SQLEi-FR194738) and synergistic regimens (apatinib +chloroquine and PI3Ki+DNMT1i). 8. Emerging strategies: Multi-node targeting (GSK3β, STAT3-YAP complex) and AI-driven dynamic network modelling for optimised combinatorial therapy.

The malignant phenotype of OS is sustained by aberrant activation or suppression of multiple oncogenic signalling pathways that collectively regulate cell proliferation, metabolic reprogramming, metastasis, immune evasion, and therapeutic resistance. Rather than operating in isolation, these pathways form a highly interconnected signalling network, with several central hubs integrating upstream oncogenic cues and downstream phenotypic outputs (**Figure 3**).

Among these pathways, the PI3K/AKT/mTOR axis functions as a core signalling hub in OS, integrating upstream inputs from receptor tyrosine kinases (RTKs), including VEGFR2, as well as mechanotransduction and cytoskeletal signals mediated by the RhoA/YAP pathway. Activation of this axis drives uncontrolled proliferation, enhances glycolytic metabolism through upregulation of LDHA and PFKM, and promotes apoptosis resistance via BCL-2 signalling, thereby supporting tumour growth and survival under therapeutic stress (42–45). Beyond these tumour-intrinsic roles, aberrant PI3K/AKT/mTOR signalling promotes immune evasion by upregulating PD-L1, increasing secretion of immunosuppressive cytokines such as IL-10 and TGF-β, downregulating MHC class I and co-stimulatory molecules, and fostering exclusion of effector T cells while attracting immunosuppressive myeloid populations (46). Moreover, this pathway functions as a key node in metabolic–immune crosstalk, linking oncogenic metabolism to immune regulation within the tumour microenvironment. Hyperactivation enhances tumour glycolysis and anabolic biosynthesis, creating a nutrient-depleted, metabolite-rich TME that impairs effector T-cell proliferation and expands regulatory T cells, while also promoting M2-like macrophage polarization, collectively reinforcing immunosuppression and resistance to checkpoint blockade. These dual roles highlight the therapeutic potential of targeting PI3K/AKT/mTOR to reprogram both tumour and immune cell metabolism and enhance anti-tumour immunity (47).

The Wnt/β-catenin pathway exhibits extensive crosstalk with PI3K/AKT/mTOR signalling, forming a positive feedback loop that amplifies oncogenic output. WNT5B activates SOX2 expression via ROR1 and simultaneously stimulates mTOR activity, reinforcing stemness and proliferative capacity. In parallel, β-catenin nuclear accumulation is regulated by GSK3β phosphorylation, which is inhibited at Ser9 by STAT3 signalling, thereby establishing a STAT3–Wnt–PI3K oncogenic synergy that promotes tumour progression and resistance (48, 49). Moreover, activation of canonical Wnt/β-catenin signalling in tumour cells drives transcriptional programs that suppress dendritic cell recruitment and function, elevate populations of regulatory T cells (Tregs), myeloid-derived suppressor cells (MDSCs) and M2-like tumour-associated macrophages, and attenuate effector CD8^+^ T-cell infiltration and activity, thereby reinforcing immune desertification and evasion (50, 51).

STAT3 serves as a critical transcriptional integrator linking inflammatory signals, growth factor signalling, and cell cycle regulation. Activated STAT3 directly induces Cyclin D1 and c-Myc to accelerate cell cycle progression, suppresses DDIT4 to relieve inhibitory constraints on mTOR signalling, and cooperates with BCL-2 to establish a potent anti-apoptotic axis. This pathway is jointly regulated by VEGFR2 and IL-6/JAK2 signalling, thereby linking angiogenesis, inflammation, and tumour cell survival (52–56). On the other hand, persistent oncogenic STAT3 activity contribute to tumour stroma organization and extracellular matrix (ECM) remodelling, which constitute physical and biochemical barriers to effective antitumour immunity. STAT3 signalling within cancer-associated fibroblasts (CAFs) drives fibrotic ECM deposition, increases stromal stiffness, and creates an immunosuppressive niche that impedes T-cell infiltration and limits antibody penetration. Aberrant STAT3 activity enhances expression of matrix-remodelling enzymes and pro-fibrotic cytokines, reinforcing a hostile TME for immune effector cells (57).

TGF-β signalling displays a context-dependent dual role during OS progression. In early tumourigenesis, TGF-β exerts tumour-suppressive effects by activating SMAD-dependent signalling, inducing cyclin-dependent kinase inhibitors such as p15INK4B and p21CIP1, and triggering apoptosis. However, during advanced stages, dysregulation of pathway components results in a functional switch, enabling TGF-β to promote tumour growth, EMT, and metastasis. Through coordinated activation of SMAD-dependent and SMAD-independent pathways, TGF-β upregulates matrix metalloproteinases, suppresses E-cadherin, and induces mesenchymal markers including N-cadherin, vimentin, and fibronectin, thereby facilitating invasion and lung metastasis. Moreover, TGF-β enhances chemoresistance by inducing multidrug resistance proteins, activating PI3K/AKT signalling, and suppressing apoptosis, while concurrently promoting immune evasion by inhibiting NK cell activity, impairing dendritic cell maturation, reducing T-cell cytotoxicity, and inducing PD-L1 expression (58–60).

Multiple signalling pathways converge on the regulation of autophagy–apoptosis balance, which critically determines therapeutic response. The STAT3/BCL-2 axis suppresses Beclin-1-dependent autophagy, whereas inhibition of VEGFR2/STAT3 signalling by apatinib relieves BCL-2-mediated autophagy repression and concurrently activates caspase-dependent apoptosis. These findings underscore the adaptive survival mechanisms that limit single-agent efficacy (61).

Based on this interconnected signalling architecture, preclinical studies have explored combination strategies targeting multiple nodes, including ROR1 (D10), STAT3 (T52), mTOR (rapamycin analogues), and SQLE (FR194738). Notably, apatinib combined with the autophagy inhibitor chloroquine effectively overcomes compensatory survival pathways and markedly enhances antitumour efficacy (48, 61, 62). These results highlight the necessity of network-oriented therapeutic approaches, targeting shared signalling hubs such as GSK3β or STAT–YAP complexes, and support the application of artificial intelligence-assisted modelling to design rational multi-target combinations, including signalling–epigenetic co-inhibition strategies.

Additional pathway-specific regulators further modulate therapeutic sensitivity. ZIP10 enhances tumour proliferation and cisplatin resistance via activation of the CREB–ITGA10–PI3K/AKT axis, whereas SQLE-driven cholesterol metabolism activates the FAK/PI3K/AKT/mTOR pathway. Inhibition of SQLE with FR194738, combined with cisplatin, significantly suppresses tumour growth *in vivo* (63, 64). Conversely, RILP suppresses PI3K/AKT/mTOR signalling and induces autophagy-dependent apoptosis by binding Grb10, while HMGCL promotes autophagy and inhibits tumour progression via β-hydroxybutyrate-mediated pathway modulation; accordingly, the HMGCR-targeting agent simvastatin is currently undergoing preclinical validation (65, 66).

Translationally, the PI3K/mTOR dual inhibitor samotolisib is being evaluated in combination with chemotherapy in an ongoing clinical trial (NCT03213678), offering new therapeutic options for advanced OS. The Wnt/β-catenin pathway also drives epithelial–mesenchymal transition (EMT) and lung metastasis through activation of WNT5A signalling via the TSPAN9–integrin β1–FAK–Ras–ERK1/2 axis. Small-molecule inhibition of the WNT5A–PI3K/AKT axis by B2 suppresses tumour growth, while PDGFD inhibits EMT by repressing MMP9 and β-catenin/ZEB signalling, suggesting potential combinatorial immunotherapeutic strategies (67–69). Moreover, a Phase II clinical trial of the oral Wnt inhibitor LGK975 (NCT01351103) further supports the translational relevance of Wnt-targeted intervention.

Overall, the malignant behaviour of OS is orchestrated by highly interconnected signalling networks governing proliferation, metabolism, and survival. These pathways operate in close concert with the tumour microenvironment, which modulates immune responses, metastatic potential, and therapeutic resistance. Integrating insights from both signalling dysregulation and TME dynamics is therefore essential for developing more effective treatment strategies.

## 5 Tumour microenvironment in OS

The tumour microenvironment (TME) plays a pivotal role in OS progression by orchestrating complex cellular interactions, immune regulation, and metabolic adaptation. Rather than serving as a passive bystander, the OS TME actively cooperates with tumour-intrinsic genomic, epigenetic, and signalling abnormalities to promote tumour growth, metastasis, immune evasion, and therapeutic resistance.

**Figure 4:**
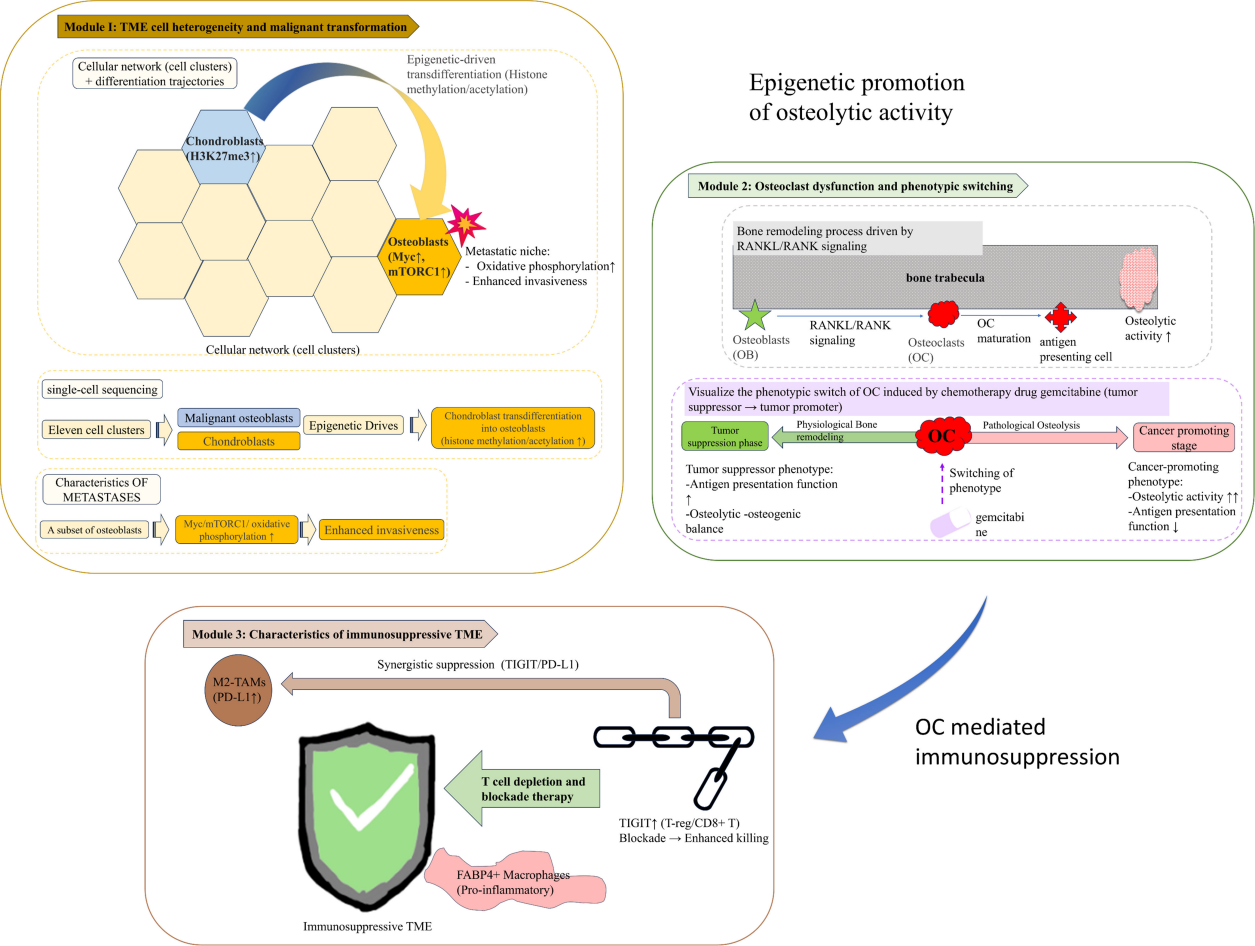
Tumour microenvironment (TME) dynamics and immune suppression in osteosarcoma. Module 1. Cellular Heterogeneity and Malignant Transdifferentiation: Single-cell sequencing identifies 11 distinct cellular clusters within the osteosarcoma TME, including malignant osteoblasts (MYC↑, mTORC1↑, oxidative phosphorylation↑) and chondroblasts (H3K27me3↑). Epigenetically driven trans-differentiation promotes chondroblast-to-osteoblast conversion. Metastatic osteoblast subclusters exhibit enhanced invasiveness via oxidative phosphorylation. Module 2. Osteoclast (OC) Dysfunction and Phenotypic Switch. RANKL/RANK signalling mediates osteoclast maturation, which is regulated by osteoblast activity. Chemotherapy induces OC phenotypic switching from tumour-suppressive (antigen presentation II) to pro-tumorigenic (osteolytic activity III and antigen presentation IV) phenotypes. Loss of osteoclast–osteoblast coupling exacerbates pathological bone resorption. Module 3. Immunosuppressive TME Features: M2-polarized tumour-associated macrophages (M2-TAMs) dominate the immune system. FABP4+ alveolar macrophages drive proinflammatory signalling in lung metastatic niches. T cell exhaustion is marked by TIGIT upregulation in T regulatory and CD8+ T cells. TIGIT blockade restores antitumour cytotoxicity. Dashed grey arrows indicate synergistic immunosuppression via TIGIT/PD-L1 crosstalk.

**Figure 5:**
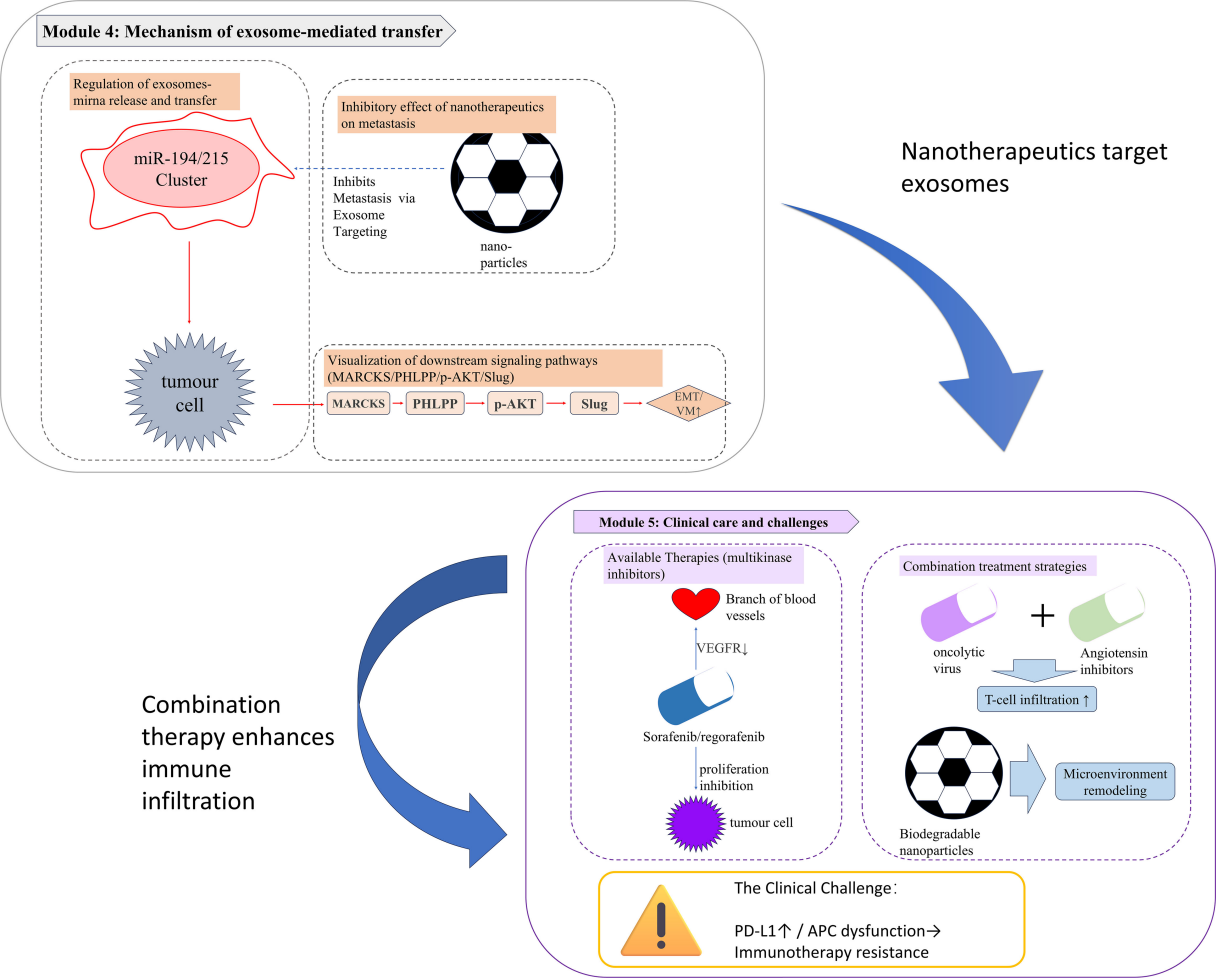
Exosome-mediated metastatic mechanisms and therapeutic challenges in osteosarcoma. Module 1. Exosome-Driven Metastatic Cascade: Tumour-derived exosomes carrying miR-194/215 clusters were released into the extracellular matrix and internalised by recipient cells. These miRNAs activate the MARCKS/PHLPP/p-AKT/Slug pathway, driving epithelial–mesenchymal transition (EMT) and vasculogenic mimicry (VM), which are key processes in metastatic dissemination. Bioengineered nanoparticles functionalized with exosome-targeting ligands inhibit metastasis by blocking exosomal miRNA delivery. Module 2. Clinical Strategies and Microenvironmental Barriers: Current therapies: Multi-kinase inhibitors (e.g., sorafenib, regorafenib, and blue pills) suppress tumour proliferation and angiogenesis via VEGFR inhibition. Emerging combinations: Oncolytic viruses combined with angiotensin inhibitors enhance T cell infiltration. Biodegradable nanoparticles remodel the immunosuppressive TME. Key challenges: Immune evasion driven by PD-L1 upregulation and antigen-presenting cell (APC) dysfunction (warning icon).

Single-cell RNA sequencing has revealed pronounced cellular heterogeneity within OS lesions, identifying at least 11 major cell clusters, including malignant osteoblasts, chondroblasts, immune cells, endothelial cells, and stromal populations (**Figures 4**, **5**). Trajectory analyses indicate that malignant chondroblasts can differentiate toward osteoblastic lineages, a process likely driven by epigenetic reprogramming mechanisms such as histone methylation and acetylation. This lineage plasticity provides tumour cells with adaptive advantages under environmental and therapeutic stress. Importantly, osteoblast-like tumour cells at metastatic sites exhibit a more aggressive phenotype than those in primary lesions. These metastatic osteoblast subsets display elevated activation of MYC, mTORC1, and oxidative phosphorylation pathways, reflecting enhanced metabolic capacity and proliferative fitness that support metastatic colonisation and outgrowth (70, 71). These findings suggest that epigenetically driven lineage switching and metabolic reprogramming jointly contribute to tumour aggressiveness during OS progression.

Bone-resorbing osteoclasts (OCs) are integral components of the OS TME and participate in pathological bone remodelling through RANKL/RANK signalling. Osteoclast maturation is tightly regulated by osteoblast activity and can be further influenced by chemotherapeutic agents such as gemcitabine. Notably, mature OCs undergo a functional shift characterised by reduced antigen-presenting capacity and enhanced osteolytic activity, indicating a transition from a tumour-suppressive to a tumour-promoting phenotype (71, 72). This phenotypic switch facilitates bone destruction and tumour expansion while concurrently weakening local immune surveillance.

Tumour-associated macrophages (TAMs) represent another dominant immunomodulatory population in the OS TME. The immunosuppressive milieu of primary OS lesions is characterised by M2-like TAMs expressing high levels of T cell exhaustion markers, including TIGIT, whereas lung metastases are enriched in FABP4^+^ alveolar macrophages with proinflammatory but tumour-supportive properties (70, 73, 74). These macrophage subsets dynamically interact with tumour cells and stromal components, shaping immune tolerance and metastatic niches. Emerging evidence indicates that TAMs actively transmit pro-metastatic signals through extracellular vesicles. For example, M2 macrophages deliver PDE4C mRNA to tumour cells via exosomes, activating collagen-related pathways (COL11A2 and COL9A3), promoting metastasis, and suppressing CD8^+^ T cell infiltration. These findings highlight the therapeutic potential of targeting TAM-derived signalling pathways, including PDE4C, to disrupt tumour–macrophage crosstalk and restore antitumour immunity (75).

The OS TME is profoundly immunosuppressive and represents a major barrier to effective immunotherapy. Single-cell studies consistently demonstrate enrichment of immunodepleted T cell populations, including PD-1^+^ and TIGIT^+^ CD8^+^ T cells, regulatory T cells (Tregs), and M2-like TAMs, particularly within lung metastases, where the expression of immunosuppressive molecules is significantly upregulated (71, 76, 77).

Although immune checkpoint inhibitors (ICIs) have achieved remarkable success in haematologic malignancies, their efficacy in OS remains limited, with response rates to PD-1/PD-L1 monotherapy below 20% (78). Several intrinsic biological barriers underlie this limited activity, including low tumour antigenicity, physical exclusion imposed by the dense, mineralized osteoid matrix, and a profoundly immunosuppressive, myeloid-dominated tumour microenvironment.

One fundamental limitation of immune checkpoint blockade in OS is its relatively low tumour mutational burden and limited neoantigen landscape. Although OS is characterized by extensive structural genomic alterations, including copy number variations and chromothripsis, these events do not necessarily translate into a high load of immunogenic neoantigens. Consequently, insufficient priming of tumour-specific cytotoxic T lymphocytes restricts the efficacy of PD-1/PD-L1 blockade, which critically depends on pre-existing antitumour immunity (79, 80).

In addition to immunological constraints, the unique histopathological architecture of OS imposes a physical barrier to effective immunotherapy. The dense, mineralized osteoid matrix and aberrant bone remodeling restrict immune cell trafficking and limit intratumoural penetration of therapeutic antibodies. This physical exclusion further contributes to the immune-desert or immune-excluded phenotypes frequently observed in OS, thereby diminishing the clinical impact of immune checkpoint inhibitors (81, 82).

Moreover, OS is characterized by a profoundly immunosuppressive myeloid-dominated tumour microenvironment. Tumour-associated macrophages with an M2-like phenotype constitute a major immune cell population, producing immunosuppressive cytokines such as IL-10 and TGF-β, expressing immune checkpoints, and promoting T-cell exhaustion. These macrophage-driven networks not only blunt cytotoxic T-cell activity but also establish a self-reinforcing immunosuppressive niche that limits responsiveness to PD-1/PD-L1 monotherapy (83).

TIGIT has emerged as a promising next-generation immune checkpoint target in OS. TIGIT is broadly expressed on Tregs, CD8^+^ T cells, and natural killer (NK) cells, and its blockade significantly enhances T cell-mediated cytotoxicity against OS cells in preclinical models (71, 84). These findings provide a strong rationale for developing TIGIT-based immunotherapeutic strategies, either alone or in combination with PD-1/PD-L1 blockade.

Cell-based immunotherapies are also being actively explored. While chimeric antigen receptor T (CAR-T) cell therapy has not yet replicated its success in solid tumours such as OS, CAR-T cells targeting HER2, GD2, and other antigens have advanced to phase II clinical trials (85, 86). In parallel, CAR-NK and CAR-macrophage (CAR-M) therapies have demonstrated preclinical promise by amplifying innate immune responses (76).

Given the multifaceted immunosuppressive landscape of the OS TME, combination strategies aimed at microenvironment remodelling have become a central focus of translational research. Dual immune checkpoint blockade, such as anti-PD-L1 combined with anti-CTLA-4 or anti-TIGIT therapy, has shown improved objective response rates in preclinical settings. In addition, inhibition of indoleamine 2, 3-dioxygenase (IDO) disrupts tryptophan metabolism and mitigates Treg-mediated immunosuppression, providing a complementary approach to enhance ICI efficacy (78, 84).

Epigenetic modulation represents another promising strategy to sensitise OS to immunotherapy. Histone methylation inhibitors can induce immunogenic tumour cell death, enhance antigen presentation, and synergise with ICIs to reshape the immune microenvironment (87). Moreover, metabolic–immune crosstalk has emerged as a critical determinant of immune responsiveness. Elevated activity of vitamin and cofactor metabolic pathways is associated with favourable prognosis, and members of the apolipoprotein family (APOA1 and APOC1) influence immune cell infiltration by regulating fat-soluble vitamin transport within the TME (77).

Conversely, metabolic enzymes such as ST3GAL4 promote immune escape by enhancing glycolysis and driving macrophage M2 polarisation. Inhibition of ST3GAL4 has been shown to reverse the immunologically “cold” microenvironment, restoring antitumour immunity (77). In addition, dual blockade of the mTOR–MYC axis, for example using PI3K/mTOR inhibitors such as buparlisib, can synergistically enhance the efficacy of immune checkpoint blockade (84).

In summary, the TME of OS is shaped by dynamic interactions among malignant cells, bone-resident cells, immune populations, and metabolic cues. Dissecting cell subpopulation dynamics, efferocytosis, and exosome-mediated “self-seeding” mechanisms provides new opportunities for precision therapies targeting MerTK, immune checkpoints, and microenvironmental remodelling (70, 71, 88). Overcoming the immunosuppressive TME remains a central challenge in OS treatment, and rational combination strategies integrating immunotherapy, epigenetic modulation, and metabolic intervention are likely to be essential for improving clinical outcomes.

## 6 Emerging technologies and multimodal strategies enabling precision therapy in OS

Recent advances in nanotechnology, biomaterials science, and systems-level therapeutic design have expanded preclinical research strategies for OS treatment. To date, most of these approaches remain at an exploratory or early preclinical stage, with limited validation in clinical settings. Rather than functioning solely as drug carriers, these emerging platforms are being designed to integrate tumour ablation, immune modulation, metabolic intervention, and skeletal regeneration within experimental models, thereby providing conceptual support for future precision multimodal therapies, pending rigorous evaluation of safety, efficacy, and translatability in humans.

### 6.1 Nanotechnology-driven remodelling of the tumour microenvironment

Intelligent and stimuli-responsive nanodelivery systems have been developed to overcome the immunosuppressive and metabolically hostile TME. Mitochondria-targeting polymer micelles, such as OPDEA-PDCA, inhibit pyruvate dehydrogenase kinase to induce mitochondrial oxidative stress and tumour cell pyroptosis, while promoting soluble PD-L1 release that enhances responsiveness to immune checkpoint blockade (89). Similarly, CaCO_3_/polydopamine-based nanosystems neutralize tumour acidity and suppress lactate production, thereby reversing metabolic immunosuppression and synergizing with PD-1/PD-L1 inhibitors to induce durable systemic antitumour immunity (90).

Beyond metabolic regulation, logic-gated nanomaterials capable of sensing hypoxia, acidity, or ultrasonic stimulation have been engineered to trigger immunogenic cell death programs, including PANoptosis. Acoustic–dynamic platforms based on SrTiO_3_ exemplify this approach by coupling local tumour eradication with immune activation and bone regeneration, highlighting the feasibility of local–systemic therapeutic coordination (87).

### 6.2 Biomaterial platforms integrating tumour ablation and bone regeneration

Multifunctional biomaterials represent a critical advance toward structural and functional recovery following tumour clearance. Three-dimensional printed titanium scaffolds incorporating microwave-responsive ZIF-8 nanocomponents enable synergistic hyperthermia and chemotherapy, inducing immunogenic tumour cell death while promoting osteogenic differentiation through controlled zinc ion release (91). Mechanically adaptive nanocolumn architectures further enhance scaffold–bone integration and reduce postoperative fragility.

Hydrogel-based systems with stiffness gradients approximating native bone mechanics (2–4 GPa) have also been developed to support load-bearing reconstruction while serving as local delivery depots for immunomodulatory or epigenetic agents. Such platforms bridge oncologic therapy with biomechanical restoration, addressing a critical unmet need in OS management.

### 6.3 Immune- and metabolism-oriented multimodal nanotherapies

Targeted modulation of immune and metabolic pathways through nanotechnology has emerged as a promising strategy to convert immunologically “cold” OS tumours into “hot” lesions. Hyaluronic acid–targeted ZIF-8 nanoplatforms enabling pH-responsive gemcitabine release while delivering the IDO inhibitor D-1-MT effectively reduce MDSC infiltration and enhance CD8^+^ T cell activation (92). These systems exemplify rational co-delivery strategies aimed at overcoming myeloid-driven immunosuppression.

Radionuclide-based nanotherapies further expand the therapeutic landscape for bone-targeted malignancies. Pluronic-[²²³Ra]RaCl_2_ nanomicelles significantly enhance intracellular radionuclide delivery and DNA double-strand break induction compared with free radionuclide formulations. Surface functionalization using antibodies or aptamers improves tumour specificity, while enzyme-responsive nanocrystals employing collagenase-mediated matrix degradation enhance intratumoural penetration (93–95).

### 6.4 Precision delivery systems and gene-regulatory targeting

Advances in delivery precision have enabled effective targeting of tumour cells and stromal compartments. Mannose-modified layered double hydroxide nanocarriers facilitate selective siRNA delivery to OS cells, achieving enhanced gene silencing efficiency and providing a foundation for RNA-based therapeutic strategies (96). In parallel, acoustodynamic–epigenetic coupling nanoparticles capable of ultrasound-triggered histone deacetylase inhibitor release integrate immunogenic cell death induction with epigenetic reprogramming, reinforcing antitumour immunity (97).

### 6.5 Data-driven integration and AI-assisted therapeutic stratification

The convergence of artificial intelligence (AI) and multiomics analysis has accelerated the identification of actionable targets and patient stratification strategies. An AI-driven prognostic model incorporating cholesterol metabolism (SQLE), Wnt signalling, and immune-related genes enables accurate risk classification and informs rational combination therapy design. Pharmacological inhibition of SQLE suppresses tumour growth through blockade of the FAK/PI3K/AKT/mTOR axis and enhances chemotherapy sensitivity, supporting its translational potential (63). Complementarily, methylation-based ctDNA assays allow real-time monitoring of tumour clonal dynamics and minimal residual disease, enhancing prognostic stratification and adaptive therapeutic decision-making (98). Beyond individual targets, computational platforms integrating pan-cancer multiomics data and graph-based network modelling can identify tumour–microenvironment interaction modules, guiding personalized multimodal intervention strategies and optimizing combination selection (99–101).

Collectively, these advances reflect a paradigm shift in OS treatment from uniform chemotherapy-based regimens toward precision, microenvironment-oriented, and regeneration-integrated multimodal therapy. While challenges related to scalability, safety, and clinical translation remain, the rational integration of nanotechnology, immunotherapy, and AI-driven patient stratification offers a promising roadmap for improving both oncologic control and long-term functional outcomes in OS.

## 7 Translational barriers limiting clinical implementation

Despite advances in molecular characterization and therapeutic innovation, clinical translation of OS treatments faces several bottlenecks.

First, chemotherapy resistance remains prevalent. PXR activation induces drug-metabolizing enzymes and efflux transporters, accelerating chemotherapeutic clearance and reducing intracellular drug accumulation, thereby limiting the durability of conventional regimens (102). Second, intratumoural heterogeneity constrains precision treatment. Traditional histopathological subtypes provide limited guidance, while single-cell transcriptomics reveal coexisting stem-like, osteoblastic, chondrogenic, and adipogenic subsets within the same tumour, complicating target selection and contributing to variable responses (103, 104). Third, a gap exists between preclinical success and clinical translation of targeted or cell-based therapies. For instance, ALPL-1–specific CAR-T cells are effective in xenograft models, but clinical application is limited by cytokine release syndrome and potential on-target toxicity due to ALPL-1 expression in normal bone (105). Fourth, the TME hampers durable immunotherapy. M2-polarized TAMs promote collagen deposition and CD8^+^ T-cell exhaustion via PDE4C-containing exosomes, while IDO-driven tryptophan metabolism suppresses T-cell function, collectively limiting immune checkpoint blockade efficacy (75, 92). Fifth, existing prognostic and predictive models, including the AIDPI system, are largely retrospective and require validation in large-scale, multicentre prospective studies (63). Finally, nanotechnology-based delivery platforms face significant translational challenges, including off-target toxicity of carrier materials (e.g., cationic polymers), rapid clearance by the reticuloendothelial system, limited penetration into mineralized bone tissue, low drug-loading efficiency, and poorly characterized *in vivo* biodistribution (106–108). In addition, biologically derived carriers such as plant exosomes may exert unintended tumour-promoting effects (109). Additional barriers include immune incompatibility and limited persistence of adoptively transferred cells, uncertainties in long-term biocompatibility and degradation of scaffolds and nanomaterials, insufficient vascularization and neural integration in large bone constructs, and biological heterogeneity undermining predictability of immunomodulatory interventions.

To address these challenges, a systematic translational evaluation framework is needed, integrating large-animal bone-defect models, spatial multi-omics mapping, and longitudinal clinical monitoring. Such approaches are essential to validate emerging therapies and operationalize functional cure—durable tumour eradication with biomechanical and physiological restoration. Over the next decade, interdisciplinary convergence across oncology, immunology, materials science, and data-driven medicine is expected to shift OS management from broad-spectrum cytotoxic strategies to spatiotemporally precise interventions prioritizing both survival and long-term functional recovery.

## 8 Future perspectives: toward precision multimodal therapy and functional cure in OS

Despite substantial advances in molecular characterisation and therapeutic technologies, the current clinical paradigm for OS remains largely focused on short-term tumour control and radiological remission. These conventional endpoints inadequately capture the long-term challenges faced by survivors, including compromised bone integrity, mechanical fragility, persistent functional impairment, and sustained immune dysregulation. As survival improves, there is a growing need to redefine therapeutic success beyond tumour eradication alone.

In this context, the concept of a *functional cure* represents a necessary evolution in OS treatment goals. Rather than emphasising oncologic clearance in isolation, a functional cure prioritises durable disease control alongside preservation or restoration of skeletal function and overall physiological performance. This perspective shifts outcome evaluation from tumour-centred endpoints toward clinically meaningful, long-term functional benefit. Achieving such outcomes will require coordinated advances that extend beyond tumour-intrinsic targeting. Therapeutic strategies must account for residual disease, tumour–microenvironment interactions, and the biological processes that influence tissue integrity and immune competence, highlighting the importance of rational multimodal and combination approaches. Precision medicine will play an increasingly central role in guiding these integrative strategies. Biomarker-driven stratification models incorporating genomic, epigenetic, immune, and microenvironmental features have the potential to optimise patient selection and treatment intensity. The integration of single-cell and spatial omics technologies may further refine therapeutic decision-making, enabling more precise alignment between disease biology and intervention timing.

In general, OS remains a highly aggressive malignancy driven by coordinated dysregulation across genomic, epigenetic, signalling, metabolic, and microenvironmental layers. By synthesising recent advances across these domains, this review underscores the limitations of reductionist, single-pathway approaches and supports a network-oriented, integrative therapeutic paradigm. Ultimately, shifting the clinical focus from isolated tumour control toward durable restoration of structure, function, and immune competence may provide a more meaningful and sustainable framework for improving long-term outcomes in patients with OS.
